# Correction: ‘Observational study comparing heart rate in crying and non-crying but breathing infants at birth’

**DOI:** 10.1136/bmjpo-2023-001886corr1

**Published:** 2024-04-16

This article has been corrected since it was published online. In the published version of the article, the final paragraph of Results section erroneously reported a lower cumulative incidence of bradycardia and tachycardia in both the crying and the non-crying but breathing groups than what was seen in figure 3A and B. The corrected paragraph reads:

The median duration of the longest episode of bradycardia was 7.5 s (IQR (IQR) 3–16 s) and that of tachycardia 10 s (IQR 5–25 s). A total of 12/54 (22.2%) and 112/1155 (9.7%) infants were ever bradycardic by 180 s after birth, and 14/54 (25.9%) and 125/1155 (10.8%) were ever tachycardic in the non-crying and crying groups, respectively. Bradycardia and tachycardia largely affected separate infants; 10 infants were both bradycardic and tachycardic within the first 180 s after birth.

The original paragraph read:

The median duration of bradycardia was 7 s (IQR (IQR) 4–16 s) and that of tachycardia 11 s (IQR 6–25 s). A total of 6/54 (11.1%) and 48/1155 (4.2%) infants were ever bradycardic, and 6/54 (11.1%) and 71/1155 (6.1%) were ever tachycardic in the non-crying and crying groups, respectively. Bradycardia and tachycardia largely affected separate infants; only one infant was both bradycardic and tachycardic within the first 180 s after birth.

