# Supplementary material for: Observational study comparing heart rate in crying and non-crying but breathing infants at birth
Source: BMJ Paediatr Open. 2023 Apr 7;7(1):e001886. doi: 10.1136/bmjpo-2023-001886 (PMC10083872; doi:10.1136/bmjpo-2023-001886)
Supplement: Supplementary data [file bmjpo-2023-001886supp001.pdf]

SUPPLEMENTARY FILE

Supplementary tables

Supplementary Table 1. Details of obstetric complications among crying and non-crying but breathing neonates

**Frequency of complications**	**Crying (N=1155)**	**%**	**Non crying but breathing (N=54)**	**%**
Zero	1119	96.9	50	92.6
One	35	3.0	4	7.4
Two	1	0.1	0	0.0
Maternal conditions				
Hypothyroidism	1	0.1	0	0.0
Diabetes Mellitus inc. gestational diabetes	0	0.0	0	0.0
Oligohydramnios	1	0.1	1	1.9
Polyhydramnios	0	0.0	0	0.0
Delivery complications				
Antepartum hemorrhage	1	0.1	0	0.0
Pre-eclampsia	1	0.1	0	0.0
Eclampsia	0	0.0	0	0.0
Intrapartum fever	1	0.1	0	0.0
Chorioamnionitis	1	0.1	0	0.0
Premature rupture of membranes	4	0.3	0	0.0
Prolonged labour	12	1.0	2	3.7
Decreased fetal movements	3	0.3	0	0.0
Breech or transverse lie	4	0.3	0	0.3
Prolapsed cord	1	0.1	0	0.0
Fetal distress	3	0.3	1	1.9
Maternal distress	1	0.1	0	0.0
Fetal/neonatal conditions				
Fetal congenital anomaly	4	0.3	0	0.0
Nuchal cord	0	0.0	0	0.0
Cephalopelvic disproportion	0	0.0	0	0.0

Supplementary Table 2. Comparison of median heart rate (bpm) between crying and non-crying but breathing neonates. P values of < 0.004 were considered significant (p=0.05/12) using individual Mann-Whitney U-tests followed by *post hoc* Bonferroni correction for multiple comparisons.

	Crying		Non-crying but breathing		
Time	Median (quartiles)	N	Median (quartiles)	N	p-value
10 sec	157 (118, 174)	171	155 (104, 178)	5	0.929
30 sec	168 (146, 182)	988	164 (140, 173)	44	0.082
45 sec	167 (149, 182)	1099	165 (138, 182)	51	0.508
60 sec	168 (151, 182)	1106	162 (149, 185)	51	0.851
75 sec	169 (154, 182)	1044	168 (150, 187)	52	0.860
90 sec	169 (154, 183)	899	164 (156, 188)	50	0.559
105 sec	169 (155, 184)	794	175 (164, 190)	46	0.037
120 sec	169 (156, 183)	695	174 (161, 191)	45	0.086
135 sec	169 (156, 183)	495	173 (153, 190)	30	0.637
150 sec	170 (156, 182)	364	172 (149, 192)	24	0.655
165 sec	169 (155, 182)	290	177 (156, 192)	18	0.224
180 sec	169 (156, 182)	210	170 (161, 195)	15	0.163

Supplementary Figure. Comparison of the first minute of action in the Helping Babies Breathe 2nd edition (left) and Essential Newborn Care 1 (right) neonatal resuscitation flow-charts.

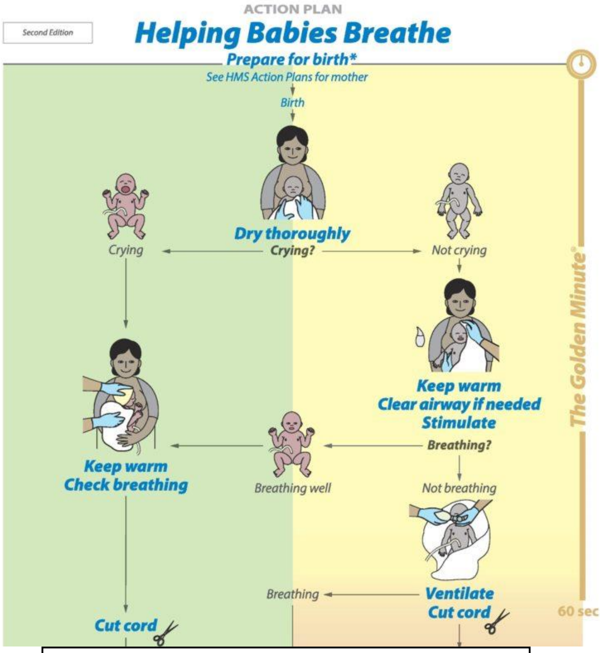

Printed with permission from Helping Babies Breathe Action Plan, 2nd edition, © 2016, American Academy of Pediatrics

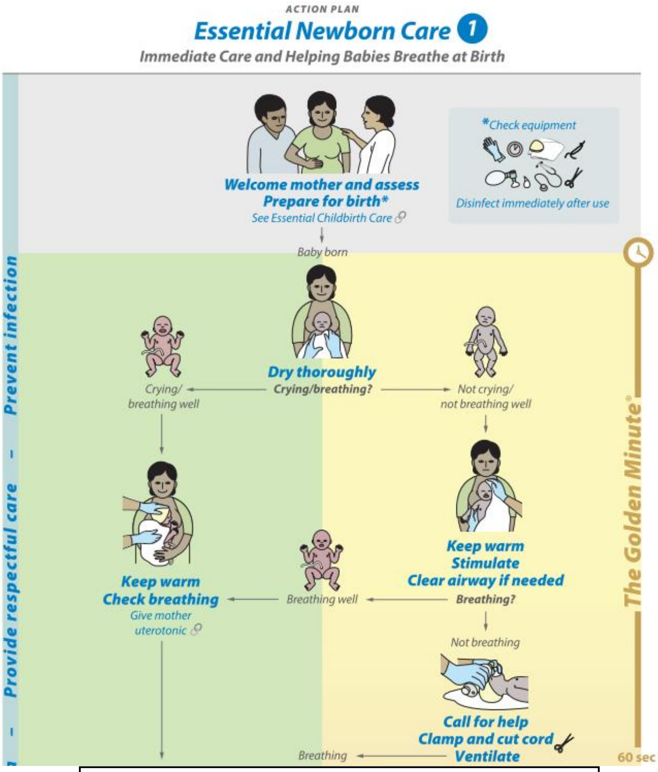

Essential Newbon Care 1 Action Plan, © 2022, World Health Organization
