# Supplementary material for: Observational study comparing heart rate in crying and non-crying but breathing infants at birth
Source: BMJ Paediatr Open. 2023 Apr 7;7(1):e001886. doi: 10.1136/bmjpo-2023-001886 (PMC10083872; doi:10.1136/bmjpo-2023-001886)
Supplement: Author's manuscript [file bmjpo-2023-001886.draft_revisions.pdf]

BMJ Paediatrics Open

BMJ Paediatrics Open is committed to open peer review. As part of this commitment we make the peer review history of every article we publish publicly available.

When an article is published we post the peer reviewers' comments and the authors' responses online. We also post the versions of the paper that were used during peer review. These are the versions that the peer review comments apply to.

The versions of the paper that follow are the versions that were submitted during the peer review process. They are not the versions of record or the final published versions. They should not be cited or distributed as the published version of this manuscript.

BMJ Paediatrics Open is an open access journal and the full, final, typeset and author-corrected version of record of the manuscript is available on our site with no access controls, subscription charges or pay-per-view fees (http://bmjpaedsopen.bmj.com).

If you have any questions on BMJ Paediatrics Open's open peer review process please email info.bmjpo@bmj.com

BMJ Paediatrics Open

Observational study comparing heart rate in crying and non-crying but breathing infants at birth

Journal:	*BMJ Paediatrics Open*
Manuscript ID	bmjpo-2023-001886
Article Type:	Original research
Date Submitted by the Author:	28-Jan-2023
Complete List of Authors:	Kukka, Antti; Uppsala universitet Disciplinary Domain of Medicine and Pharmacy, Women's and Children's Health; Region Gävleborg, Department of Pediatrics<br>Berkelhamer, Sara; University of Washington, Division of Neonatology, Department of Pediatrics,<br>Eilevstjønn, Joar; Laerdal Medical AS, Strategic Research<br>Wood, Thomas; University of Washington, Center on Human Development and Disability<br>Basnet, Omkar; Golden Community<br>KC, Ashish; Uppsala University, International Maternal and Child Health
Keywords:	Neonatology, Physiology, Resuscitation

SCHOLARONE™
Manuscripts

I, the Submitting Author has the right to grant and does grant on behalf of all authors of the Work (as defined in the below author licence), an exclusive licence and/or a non-exclusive licence for contributions from authors who are: i) UK Crown employees; ii) where BMJ has agreed a CC-BY licence shall apply, and/or iii) in accordance with the terms applicable for US Federal Government officers or employees acting as part of their official duties; on a worldwide, perpetual, irrevocable, royalty-free basis to BMJ Publishing Group Ltd ("BMJ") its licensees and where the relevant Journal is co-owned by BMJ to the co-owners of the Journal, to publish the Work in this journal and any other BMJ products and to exploit all rights, as set out in our licence.

The Submitting Author accepts and understands that any supply made under these terms is made by BMJ to the Submitting Author unless you are acting as an employee on behalf of your employer or a postgraduate student of an affiliated institution which is paying any applicable article publishing charge ("APC") for Open Access articles. Where the Submitting Author wishes to make the Work available on an Open Access basis (and intends to pay the relevant APC), the terms of reuse of such Open Access shall be governed by a Creative Commons licence – details of these licences and which Creative Commons licence will apply to this Work are set out in our licence referred to above.

Other than as permitted in any relevant BMJ Author's Self Archiving Policies, I confirm this Work has not been accepted for publication elsewhere, is not being considered for publication elsewhere and does not duplicate material already published. I confirm all authors consent to publication of this Work and authorise the granting of this licence.

Confidential: For Review Only

Observational study comparing heart rate in crying and non-crying but breathing infants at birth

Antti Kukka[1,2*] MD, MSc, Sara Berkelhamer[3*], MD, Joar Eilevstjønn[4], PhD, Thomas Ragnar Wood[5], B.M., B.Ch., PhD, Omkar Basnet[6], MSc, Ashish KC[1], MD, PhD

Affiliations:

[1]Department of Women's and Children's Health, Uppsala University, Sweden

[2]Department of Pediatrics, Region Gävleborg, Sweden

[3]Division of Neonatology, Department of Pediatrics, University of Washington, Seattle, USA

[4]Laerdal Medical, Stavanger, Norway

[5]Center on Human Development and Disability, University of Washington, Seattle, USA

[6]Golden Community, Jawagal, Nepal

*Contributed equally as co-first authors

Corresponding author: Antti Kukka, Department of Women's and Children's Health, Uppsala University, SE-751 85, Uppsala, Sweden, https://kbh.uu.se. Email address: antti.kukka@kbh.uu.se; Phone number: +46 721 811 448

Clinical trial registration number: ISRCTN18148368

Keywords: heart rate, neonatal, resuscitation

Confidential: For Review Only

Abstract

Background: Stimulating infants to elicit a cry at birth is common but could result in unnecessary handling. We evaluated heart rate in infants who were crying versus non-crying but breathing immediately after birth.

Methods: This was single-center observational study of singleton vaginally born infants at ≥33 weeks of gestation. Infants who were *crying* or *non-crying but breathing* within 30 seconds after birth were included. Background demographic data and delivery room events were recorded using tablet-based applications and synchronized with continuous heart rate data recorded by a dry-electrode electrocardiographic monitor. Heart rate centile curves for first three minutes of life were generated with piece wise regression analysis. Odds of bradycardia and tachycardia were compared using multiple logistic regression.

Results: 1155 crying and 54 non-crying but breathing neonates were included in the final analyses. There were no significant differences in the demographic and obstetric factors between the cohorts. Non-crying but breathing infants had higher rates of early cord clamping < 60 seconds after birth (75.9% vs 46.5%) and admission to the neonatal intensive care unit (13.0% vs 4.3%). There were no significant differences in median heart rates between the cohorts. Non-crying but breathing infants had higher odds of bradycardia (heart rate < 100 bpm, adjusted odds ratio 2.64; 95% Confidence Interval 1.34-5.17) and tachycardia (adjusted odds ratio 2.86; 95% Confidence Interval 1.50-5.47).

Conclusion: Infants who are quietly breathing but do not cry after birth have an increased risk of both bradycardia and tachycardia and admission to neonatal intensive care unit.

248 words

What's Known on This Topic

Stimulation of breathing infants to elicit a cry is a common practice in newborn deliveries. However, international resuscitation algorithms disagree on whether *crying* or *breathing* should guide providing stimulation.

What This Study Adds

Non-crying but breathing infants have increased risk of bradycardia, tachycardia and neonatal intensive care admission compared to crying infants.

How this study might affect research, practice or policy

While waiting for further studies assessing the role of stimulation in non-crying but breathing infants, these newborns should be monitored carefully for clinical deterioration.

Word Count: 2499

Confidential: For Review Only

Contributors statement:

Dr. Ashish KC designed the study, implemented, supervised and carried out the study and the data collection on site, reviewed data analysis and drafted the initial manuscript.

Dr. Sara Berkelhamer designed the study, reviewed data analysis and revised and edited the manuscript.

Dr. Antti Kukka designed the study, reviewed the data analysis and drafted the initial manuscript.

Dr. Joar Eilevstjønn and Dr. Thomas Ragnar Wood extracted and analyzed the heart rate data and drafted the initial manuscript.

Omkar Basnet implemented, supervised and carried out the study and the data collection on site, and extracted and analyzed the heart rate data.

All authors approved the final manuscript as submitted and agree to be accountable for all aspects of the work.

Confidential: For Review Only

INTRODUCTION

The transition to air-breathing at birth is a seminal physiological event essential to the onset of pulmonary gas exchange and postnatal survival [1]. This transition is usually accompanied by a spontaneous or elicited cry, a reassuring and readily observable sign for both health care providers and family. The clinical implication of a breathing infant who *does not cry* during the first minute after birth warrants evaluation as the globally important Helping Babies Breathe (HBB) neonatal resuscitation algorithm utilizes lack of cry alone to direct further actions in newborn resuscitation [2].

Lung fluids play a critical role in early fetal lung development, but represent an obstacle to lung expansion and gas exchange post-delivery [3,4]. Airway liquid clearance is primarily driven by the expansion of the chest wall during inspiration [5]. As compared to quiet tidal breathing, crying similarly to grunting creates greater peak inspiratory flow and a more complex biphasic expiratory volume pattern preventing backflow of liquid into the alveoli [5,6]. This pattern is associated with optimal volume redistribution from well aerated regions to those which are not as well aerated via pendelluft flow [5]. Crying is thus better suited to both rapid aeration and maintenance of functional residual capacity than tidal breathing [5].

Failure to rapidly recruit lung tissue after birth can impair the cardiovascular transition. Shift of fluid during initial partial lung aeration causes vasodilation also in unaerated regions of the lung, a ventilation-perfusion mismatch, which results in further decrease in local pulmonary vascular resistance due to increase in arterial oxygen content [7]. With clamping of the umbilical cord and closure of right-to-left shunts, pulmonary venous return becomes the sole source of cardiac output [7]. If the cord is clamped before the lung is aerated, a fall in cardiac output can theoretically lead to bradycardia [4,8]. A vigorous infant will, however, strive to maintain cardiac output by

compensatory increase in heart rate (HR) and a trial of the so called physiologically based cord clamping did not show a reduction in the rate of bradycardia [9].

For low resource settings, simple clinical signs are needed for identifying infants in need of resuscitation. We previously demonstrated higher risk of neonatal mortality among neonates who were not crying immediately after birth [10]. This observation was particularly true for non-breathing infants, but even non-crying but breathing newborns had an increased risk of predischarge mortality when compared with crying neonates (adjusted odds ratio (aOR) 12.3; 95% confidence interval (CI), 5.8–26.1) [10]. To further investigate the contribution of cry to the transitional physiology of breathing infants, we sought to compare the HR pattern in the first three minutes in infants classified as *crying* or *non-crying but breathing* immediately after birth.

METHODS

Design

This was a secondary analysis of a single center prospective observational study, which was conducted over five months (28th May to 28th October 2019) at Western Regional Hospital, Pokhara, Nepal [11]. The hospital has approximately 6500 births annually and is the public referral hospital of the province.

Ethical approval was sought from the Nepal Health Research Council (no. 87/2018) and written informed consent obtained from the pregnant women before delivery. Declaration of Helsinki by the World Medical Association was adhered to.

Deliveries were attended by a nurse-midwife or a skilled birth attendant with on-call support from a general medical doctor, pediatrician and obstetrician when needed [12]. Providers were trained

Confidential: For Review Only

in REFINE, a HR-guided neonatal resuscitation protocol, including HBB 2nd edition supplemented by a half-day orientation on the use of continuous HR monitoring [12].

Participants

All live born vaginally delivered singleton neonates at or beyond 33 weeks of gestation or with birth weight > 2500 grams were eligible for this study.

Crying newborns were defined as those who cried within approximately 30 seconds after birth.

Non-crying but breathing newborns were defined as those who did not cry but were noted to be breathing within the same period.

All newborns who were non-crying and non-breathing after birth were excluded from this study.

Data collection and management

Research nurses not part of the clinical care team recorded delivery room observations including periods of breathing and time of cord clamping using a purpose-designed application (Liveborn, Laerdal Medical, Stavanger, Norway) [12]. Oxygen saturation data was not recorded. Crying status after birth, and maternal and neonatal demographics were recorded on a supplemental application (SUSTAIN). Cases where breathing status recorded on Liveborn application did not align with the crying status documented on SUSTAIN were reviewed by expert panel (KCA, SB, JE, OB) blinded to the HR data. Comments provided by research nurses and actions taken during resuscitation were taken into consideration. As a result of the review, four cases were re-classified from non-breathing to non-crying but breathing and 18 cases were changed to non-breathing and thereby excluded.

Following thorough drying, NeoBeat continuous HR monitor (Laerdal Global Health, Stavanger, Norway) was placed on the abdomen of the newborn [13]. NeoBeat uses a proprietary algorithm

Confidential: For Review Only

based on zero crossing count that averages HR over up to 12 RR intervals. The HR data from NeoBeat was streamed to and time-synced with data collected on the Liveborn application. Bluetooth connection issues or excessive movement of the newborn resulting in absent or highly inconsistent HR required exclusion of cases.

Information from the Liveborn and SUSTAIN apps were extracted separately and cleaned. Unique identification numbers provided for each participant facilitated synchronization of data for final analysis.

Data analysis

Demographics

For the final data analysis, we excluded participants' data when the birth was registered in the Liveborn app more than 10 seconds after actual birth, time of birth was not provided, or if there was too little (<30 seconds), too late (>60 seconds after birth) or too inconsistent HR data.

Maternal and infant demographics, mode of delivery, birth weight, small for gestational age status (birth weight <10th centile for gestational age), gestational age, sex and timing of cord clamping (early <60 seconds, late ≥60 seconds after birth) were collected from patient records. List of maternal and obstetric complications considered is presented in Supplementary Table 1.

Means in the two cohorts were compared using independent samples t-test and medians by Wilcoxon signed ranked test. Proportions were compared by Pearson's Chi-square test.

Confidential: For Review Only

Heart Rate data

HR data for the crying and non-crying but breathing groups from 10 until 180 seconds after birth were analyzed using centile curves at 3rd, 10th, 25th, 50th, 75th, 90th and 97th centile. The curves were smoothed using piecewise cubic spline fitting. Median HRs at two serial time points were compared using Mann Whitney U test with *post hoc* Bonferroni correction of p-values to adjust for repeated measures ($p=0.05/12 = p \leq 0.004$ for significance).

The OR of bradycardia and tachycardia were explored by logistic regression. Time of cord clamping, gestational age in weeks, birthweight in kilograms, and obstetric complications including abnormal fetal HR were included as covariates. Results are presented as aOR with 95% CI.

Cumulative proportion of neonates with bradycardia (HR <100 bpm for $\geq$1 seconds) and tachycardia (HR $\geq$200 bpm for $\geq$1 seconds) in the crying and non-crying but breathing groups were presented using a generalized additive model with 95% CI of continuous data recorded every second.

Analyses were performed using R version 4.1.2 (Vienna, Austria) and MATLAB R2021a (Mathworks Inc, Natick, MA).

Patient participation

No patients were involved in the design of the study.

RESULTS

During the study period, 3578 deliveries took place at the hospital of which 1028 were not assessed for eligibility. Of the 2550 births observed, Liveborn was not used in 368 neonates, 55 resulted in a fresh stillbirth and 23 neonates had a gestational age less than 33 weeks. Of the neonates observed

Confidential: For Review Only

with Liveborn, 895 were excluded due to issues with the HR data, LiveBorn application or gestational age. Among the 1209 neonates included in the final analyses, 1155 were classified as crying and 54 as non-crying but breathing immediately after birth (Fig. 1).

There was no significant difference in maternal age, parity or ethnicity in the two cohorts. Infants in the two groups had similar birth weight, gestational age, sex distribution and median Apgar scores at 1 and 5 minutes (Table 1). Obstetric complications during admission, abnormal fetal HR during labor and rates of instrumental delivery were also comparable (Supplementary Table 1).

Table 1: Demographic, obstetric and neonatal characteristics of crying and non-crying but breathing infants

**Indicators**	**Crying (N=1155)**	**Non crying but breathing (N=54)**	**p-value**
**Demographic factors**			
**Maternal Age,** mean ± SD	24.2 ± 4.3	24.1 ± 4.6	0.29
**Parity,** n (%)			0.30
Primipara	622 (53.9)	33 (61.1)	
Multipara	533 (46.1)	21 (38.9)	
**Ethnicity,** n (%)			0.45
Dalit	250 (21.6)	15 (27.8)	
Janajati	443 (38.4)	24 (44.4)	
Madhesi	13 (1.1)	0 (0.0)	
Muslim	24 (2.1)	1 (1.9)	
Brahmin/Chhetri	425 (36.8)	14 (25.9)	
Others	0 (0.0)	1 (0.9)	
**Pregnancy and delivery**			
**Obstetric Complications,** n (%)			0.09
No	1119 (96.9)	50 (92.6)	
Yes	36 (3.1)	4 (7.4)	
**Abnormal fetal HR during labor,** n (%)			0.47
No	1144 (99.0)	54 (100.0)	
Yes	11 (1.0)	0 (0.0)	

Confidential: For Review Only

**Mode of Delivery,** n (%)			0.13
Spontaneous Vaginal	1115 (96.5)	50 (92.6)	
Instrumental	40 (3.5)	4 (7.4)	
**Neonatal factors**			
**Birth Weight grams*,** mean±SD	3065.7±414.8	3047.2±410.5	0.55
**Gestational Age weeks**,** mean±SD	39.2±1.3	39.1±1.6	0.19
**Preterm <37 weeks**,** n (%)			0.56
No	1095 (96.1)	51 (94.4)	
Yes	45 (3.9)	3 (5.6)	
**Small for gestational age <10th centile ***,** n (%)			0.66
No	942 (82.8)	46 (85.2)	
Yes	195 (17.2)	8 (14.8)	
**Cord Clamping,** n (%)			<0.001
Early <60 seconds after birth	537 (46.5)	41 (75.9)	
Delayed ≥60 seconds after birth	618 (53.5)	13 (24.1)	
**Sex,** n (%)			0.86
Male	634 (54.9)	29 (53.7)	
Female	521 (45.1)	25 (46.3)	
**Apgar Score**, Median (IQR)			
At 1 min	7 (7-7)	6 (6-6)	0.46
At 5 min	8 (8-8)	7 (7-8)	0.68
Missing *=3, **15, ***19.			
Chi-square test, Independent samples t-test, non-parametric comparison of medians			

Non-crying but breathing infants had higher proportion of early cord clamping than crying infants (75.9% vs 46.5%, p<0.001) and were more often admitted to the neonatal intensive care unit (13.0% vs 4.3%, p=0.003) (Table 2).

Confidential: For Review Only

Table 2. Care of crying and non-crying but breathing infants

**Indicators**	**Crying N=1155**	**Non crying but breathing N=54**	**p-value**
**Positive Pressure Ventilation <3 min,** n (%)			0.76
No	1153 (99.8)	54 (100.0)	
Yes	2 (0.2)	0 (0.0)	
**Transfer to neonatal intensive care unit,** n (%)			0.003
No	1105 (95.7)	47 (87.0)	
Yes	50 (4.3)	7 (13.0)	
Chi-square test			

The smoothed 3rd, 10th, 25th, 50th, 75th, 90th and 97th HR centiles in crying and non-crying but breathing groups are displayed in Fig. 2(a) and (b). The 10th centile HR remained <100 bpm out to 35 and 20 seconds in the non-crying but breathing and crying cohorts, respectively. There were no significant differences in median HRs in the crying and non-crying but breathing cohorts between 10 and 180 seconds (Supplementary Table 2).

The aOR of bradycardia during the first 180 seconds after birth was increased in the non-crying but breathing cohort compared to the crying cohort (2.64; 95% CI 1.34-5.17; p=0.005). Similarly, the aOR of tachycardia was increased to 2.86 (95% CI 1.50-5.47, p=0.002) (Table 3).

Table 3. Adjusted Odds Ratio for experiencing bradycardia (heart rate <100 bpm) and tachycardia (heart rate ≥200 bpm) after cord clamping within the first 180 seconds after birth.

	**Bradycardia**	**Tachycardia**
Non-crying	**2.64 (1.34-5.17; p=0.005)**	**2.86 (1.50-5.47; p=0.002)**
Gestational age (per week)	0.98 (0.85-1.13; p=0.77)	1.06 (0.92-1.22; p=0.41)
Birthweight (per kg)	1.23 (0.77-1.99; p=0.38)	1.09 (0.69-1.73; p=0.71)
Obstetric complications	1.43 (0.73-2.80; p=0.30)	**3.43 (2.00-5.87; p<0.001)**
Early cord clamping (<60 seconds)	1.12 (0.77-1.64; p=0.55)	1.03 (0.72-1.48; p=0.86)

Confidential: For Review Only

Median (interquartile range) duration of bradycardia was 7 seconds (4-16 seconds) and tachycardia 11 seconds (6-25 seconds). A total of 6/54 (11.1%) and 48/1155 (4.2%) infants were ever bradycardic, and 6/54 (11.1%) and 71/1155 (6.1%) ever tachycardic in the crying and non-crying groups, respectively. Bradycardia and tachycardia largely affected separate infants – only one infant was both bradycardic and tachycardic within the first 180 seconds after birth.

A comparison of the cumulative proportion of infants with bradycardia and tachycardia in the crying and non-crying but breathing groups is presented Fig. 3 (a) and (b). Bradycardia appeared to be a relatively early phenomenon, with tachycardia appearing later, particularly in the non-crying but breathing group.

DISCUSSION

Crying confers physiological advantage over quiet breathing for clearance of lung liquids at birth. This single center observational study compared HR in 1155 crying and 54 non-crying but breathing neonates during the first three minutes after birth. There was no difference in the median HR between the two groups, but a larger proportion of non-crying but breathing neonates displayed unstable HR pattern of either bradycardia (aOR 2.64, 95% CI 1.34-5.17; p=0.005) or tachycardia (aOR 2.86, 95% CI 1.50-5.47, p=0.002) and higher risk of admission to neonatal intensive care unit.

Previous papers have presented centile curves for neonatal HR [9,14–16]. Crying infants were similar to term vaginally born cohort described by Bjorland et al. [15], but non-crying but breathing infants were more likely to remain bradycardic and experience delayed tachycardia. Prior studies have identified an increased risk of bradycardia among preterm neonates [14], however the distribution of gestational age and birth weight did not differ between the crying and non-crying

Confidential: For Review Only

but breathing groups in our study. Timing of cord clamping also impacts HR with early clamping increasing the risk of bradycardia [16,17]. In this study non-crying but breathing neonates were more likely to undergo early cord clamping than crying neonates (75.9% vs 46.5%), but the difference in odds of bradycardia remained statistically significant after this and other adjustments. Early cord clamping may, however, explain some of the differences in timing of bradycardia observed.

Another cause of bradycardia in newborns is hypoxia-induced vagal reflex [18]. This phenomenon has been shown most convincingly in relation to apnoea in the setting of obstructed placental blood flow [19], but as newborns who do not cry at birth have slower clearance of liquid from airways [5], it is conceivable that even non-crying but breathing neonates could have lower oxygenation than crying neonates. Unfortunately, we were not able to measure peripheral saturation during the study to verify this potential explanatory mechanism. Lack of pulse oximeters is a common limitation in low-income settings and until their availability increases, caregivers need actionable clinical signs like crying to guide their resuscitation efforts.

Results of this study have potential implications for neonatal resuscitation algorithms and clinical practice. Current resuscitation guidelines are inconsistent in whether breathing or crying should guide the need for resuscitation (Supplementary Fig 1). The HBB algorithm uses crying as an indicator that the infant is breathing well enough to not need stimulation [2]. In contrast, to avoid unnecessary handling the recently released World Health Organization Essential Newborn Care 1 algorithm suggests that if an infant is either crying or breathing well then stimulation is not required [20].

Differentiating well breathing from less effective respiratory effort is challenging and saturation measurement or abdominal breathing monitoring are not always available in low-income settings.

https://mc.manuscriptcentral.com/bmjpo

Findings of this study, along with previous research showing higher odds of mortality among non-crying but breathing infants as compared to those who were crying at birth [10], suggest that the role of stimulation in non-crying but breathing neonates be examined in a randomized trial. However, such study would be hard to conduct as the proportion of non-crying but breathing infants is low.

Strengths

Outcome data of the study was measured with NeoBeat HR monitor that has been shown to record efficient and reliable HR data [13]. Trained external data collectors directly observed all births and classified crying status immediately after stabilization [11,12,21]. To further increase reliability of the crying classification, we cross-tabulated data from breathing observations made real time during the initial stabilization and found high rates of agreement. Any potential misclassification might nonetheless have created spurious associations as the number of non-crying but breathing infants was relatively small.

Limitations

Evaluating breathing patterns in a newborn can be difficult and complex systems utilizing flow measurement, video and audio recordings are required for confident classification [5]. In this study, crying and breathing status at birth were determined based on overall impression during the first 30 seconds after birth with unclear interobserver reliability and risk of bias compared with video recording. The results should therefore be considered preliminary and advocate for validation with a video review. We were also unable to separate any form of breathing from breathing well, which might have biased the non-crying but breathing cohort towards sicker babies than what is intended with the Essential Newborn Care 1 resuscitation algorithm [20]. The same

Confidential: For Review Only

classification issue is, however, encountered by practitioners trying to apply the algorithm to clinical practice. Exclusion of circa 15 % of babies born with caesarean section limits the generalizability of the findings [12]. This study was underpowered to explore differences in outcome beyond admission to neonatal intensive care unit. Lastly, the potential relationship between non-crying and hypoxia as the explanatory mechanism for bradycardia and tachycardia could not be defined due to lack of concomitant oxygen saturation measurement. Further studies allowing recorded review for classification of both crying and breathing status coupled with simultaneous HR and saturation measurement should be performed in a sufficiently large population group to provide definitive guidance.

CONCLUSIONS

Crying is a simple clinical indicator of likely adequate lung recruitment facilitating normal physiologic transition at birth. Infants who are breathing but *do not cry* are at increased risk of cardiopulmonary instability and should therefore be monitored closely.

https://mc.manuscriptcentral.com/bmjpo

Confidential: For Review Only

Acknowledgements: We would like to thank research nurses, pediatricians and nurses in labor and delivery unit at Pokhara Academy of Health Sciences. We would like to thank Ram Chandra Bastola and Pratiksha Bhattarai for the clinical advice and Nick Brown for his comments on the manuscript.

Funding: Laerdal Foundation for Acute Medicine, Norway (2019-40499) and Grand Challenges Canada (1910-30925).

Role of funder: The external funding sources had no role in study design, data collection, data analysis, data interpretation, writing of the report or in the decision to submit the paper for publication.

Conflict of interest: JE is employed at Laerdal Medical. AK received PhD funding via grant Laerdal Foundation grant given to Uppsala University. All other authors had no other financial relationships with any organizations that might have an interest in the submitted work in the previous 3 years or no other relationships or activities that could appear to have influenced the submitted work.

Confidential: For Review Only

REFERENCES

1 Hooper SB, Polglase GR, Roehr CC. Cardiopulmonary changes with aeration of the newborn lung. *Paediatric respiratory reviews* 2015;**16**:147–50. doi:10.1016/j.prrv.2015.03.003

2 Kamath-Rayne BD, Thukral A, Visick MK, *et al.* Helping Babies Breathe, Second Edition: A Model for Strengthening Educational Programs to Increase Global Newborn Survival. *Global Health: Science and Practice* 2018;**6**:538–51. doi:10.9745/GHSP-D-18-00147

3 Gao Y, Raj JU. Regulation of the pulmonary circulation in the fetus and newborn. *Physiological reviews* 2010;**90**:1291–335. doi:10.1152/physrev.00032.2009

4 Hooper SB, Polglase GR, Te Pas AB. A physiological approach to the timing of umbilical cord clamping at birth. *Archives of Disease in Childhood-Fetal and Neonatal Edition* 2015;**100**:F355–60. doi:10.1136/archdischild-2013-305703

5 Tingay DG, Farrell O, Thomson J, *et al.* Imaging the Respiratory Transition at Birth: Unraveling the Complexities of the First Breaths of Life. *Am J Respir Crit Care Med* 2021;**204**:82–91. doi:10/gn7nws

6 te Pas AB, Wong C, Kamlin COF, *et al.* Breathing patterns in preterm and term infants immediately after birth. *Pediatric Research* 2009;**65**:352–6. doi:10.1203/PDR.0b013e318193f117

7 Hooper SB, Roberts C, Dekker J, *et al.* Issues in cardiopulmonary transition at birth. *Seminars in Fetal and Neonatal Medicine* 2019;**24**:101033. doi:10.1016/j.siny.2019.101033

8 Bhatt S, Alison BJ, Wallace EM, *et al.* Delaying cord clamping until ventilation onset improves cardiovascular function at birth in preterm lambs. *The Journal of physiology* 2013;**591**:2113–26. doi:10.1113/jphysiol.2012.250084

9 Badurdeen S, Davis PG, Hooper SB, *et al.* Physiologically based cord clamping for infants ≥32+0 weeks gestation: A randomised clinical trial and reference percentiles for heart rate and oxygen saturation for infants ≥35+0 weeks gestation. *PLoS Med* 2022;**19**:e1004029. doi:10.1371/journal.pmed.1004029

10 Ashish KC, Lawn JE, Zhou H, *et al.* Not Crying After Birth as a Predictor of Not Breathing. *Pediatrics* 2020;**145**:e20192719. doi:10.1542/peds.2019-2719

11 Gurung R, Gurung A, Basnet O, *et al.* REFINE (Rapid Feedback for quality Improvement in Neonatal rEsuscitation): an observational study of neonatal resuscitation training and practice in a tertiary hospital in Nepal. *BMC Pregnancy Childbirth* 2020;**20**:1–6. doi:10.1186/s12884-020-03456-z

12 Bhattarai P, Gurung R, Basnet O, *et al.* Implementing quality improvement intervention to improve intrapartum fetal heart rate monitoring during COVID-19 pandemic-observational study. *PloS one* 2022;**17**:e0275801. doi:10.1371/journal.pone.0275801

Confidential: For Review Only

13 Pike H, Eilevstjønn J, Bjorland P, *et al.* Heart rate detection properties of dry-electrode ECG compared to conventional 3-lead gel-electrode ECG in newborns. *BMC research notes* 2021;**14**:1–5. doi:10.1186/s13104-021-05576-x

14 Dawson JA, Kamlin COF, Wong C, *et al.* Changes in heart rate in the first minutes after birth. *Archives of Disease in Childhood-Fetal and Neonatal Edition* 2010;**95**:F177–81. doi:10.1136/adc.2009.169102

15 Bjorland PA, Ersdal HL, Eilevstjønn J, *et al.* Changes in heart rate from 5 s to 5 min after birth in vaginally delivered term newborns with delayed cord clamping. *Archives of Disease in Childhood - Fetal and Neonatal Edition* Published Online First: 10 November 2020. doi:10.1136/archdischild-2020-320179

16 Padilla-Sánchez C, Baixauli-Alacreu S, Cañada-Martínez AJ, *et al.* Delayed vs immediate cord clamping changes oxygen saturation and heart rate patterns in the first minutes after birth. *The Journal of Pediatrics* 2020;**227**:149-156. e1. doi:10.1016/j.jpeds.2020.07.045

17 Kc A, Kong SYJ, Haaland SH, *et al.* Increased risk of bradycardia in vigorous infants receiving early as compared to delayed cord clamping at birth. *J Perinatol* Published Online First: 31 December 2022. doi:10.1038/s41372-022-01593-1

18 Te Pas AB, Sobotka K, Hooper SB. Novel Approaches to Neonatal Resuscitation and the Impact on Birth Asphyxia. *Clinics in Perinatology* 2016;**43**:455–67. doi:10.1016/j.clp.2016.04.005

19 Dawes GS, Jacobson HN, Mott JC, *et al.* The treatment of asphyxiated, mature foetal lambs and rhesus monkeys with intravenous glucose and sodium carbonate. *The Journal of physiology* 1963;**169**:167–84. doi:10.1113/jphysiol.1963.sp007248

20 World Health Organization. WHO recommendations on newborn health: guidelines approved by the WHO Guidelines Review Committee. 2017.https://apps.who.int/iris/rest/bitstreams/1090525/retrieve (accessed 9 Jun 2022).

21 and EN-BIRTH Study Group, Kc A, Peven K, *et al.* Neonatal resuscitation: EN-BIRTH multi-country validation study. *BMC Pregnancy Childbirth* 2021;**21**:235. doi:10.1186/s12884-020-03422-9

Confidential: For Review Only

Supplementary material

Supplementary Table 1. Details of obstetric complications among crying and non-crying but breathing neonates

Supplementary Table 2. Comparison of median heart rate (bpm) between crying and non-crying but breathing neonates. P values of < 0.004 were considered significant (p=0.05/12) using individual Mann-Whitney U-tests followed by *post hoc* Bonferroni correction for multiple comparisons.

Supplementary Figure: Comparison of the Helping Babies Breathe 2nd edition and the Essential Newborn Care Now resuscitation algorithms.

Confidential: For Review Only

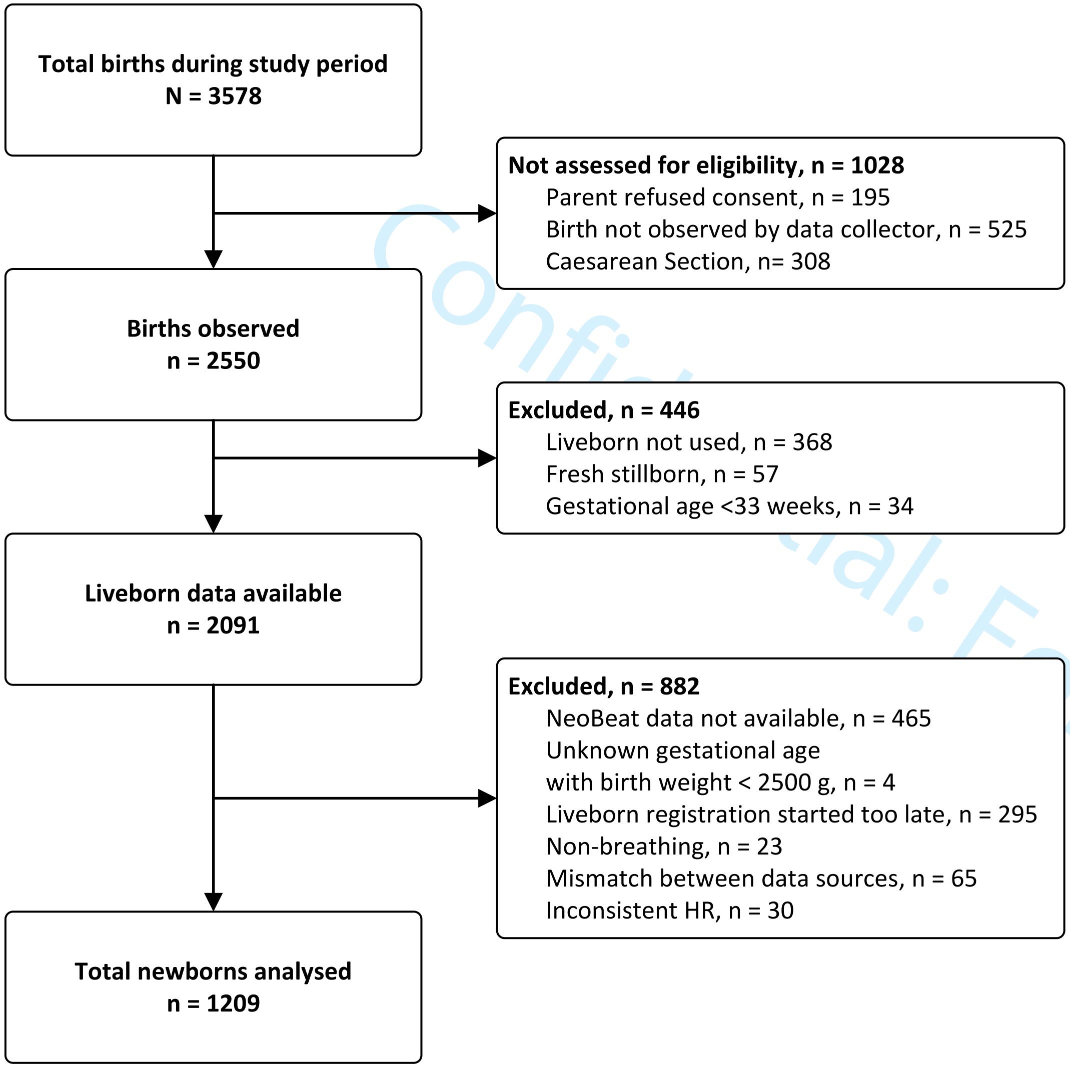

Confidential: For Review Only

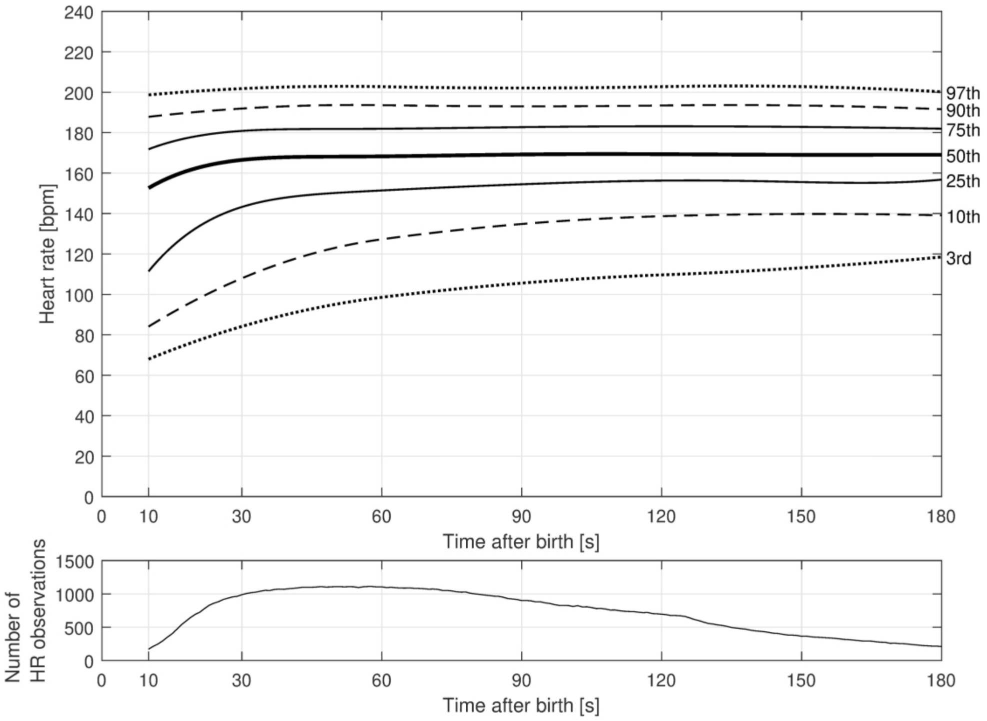

Figure 2a: Smoothed heart rate percentiles for crying infants during the first 180 seconds after birth

198x148mm (300 x 300 DPI)

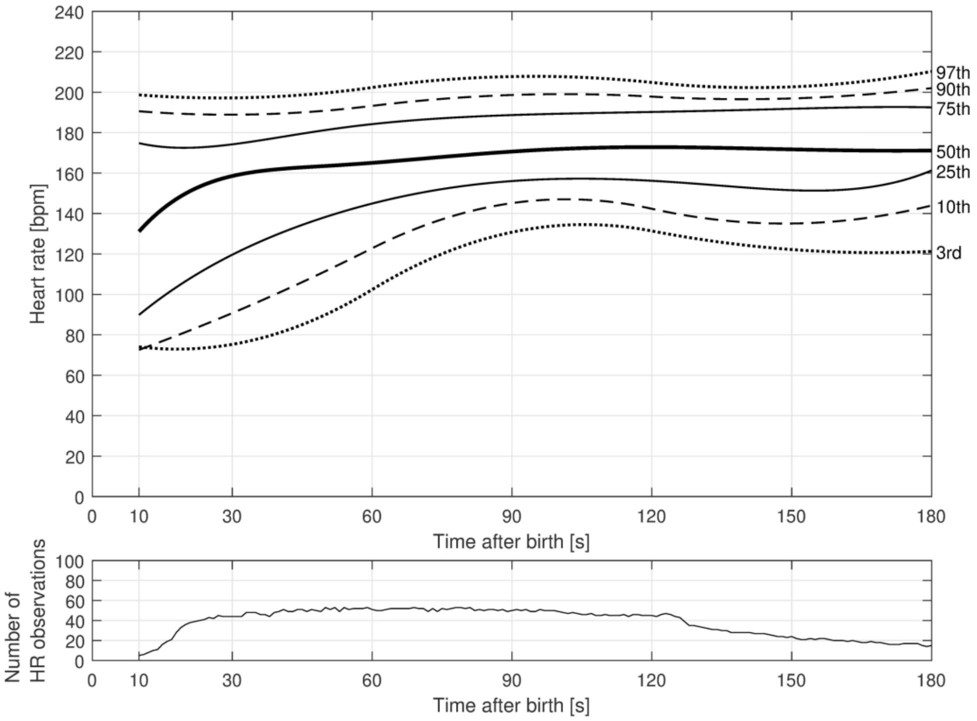

Figure 2b: Smoothed heart rate percentiles for non-crying but breathing infants during the first 180 seconds after birth

198x148mm (300 x 300 DPI)

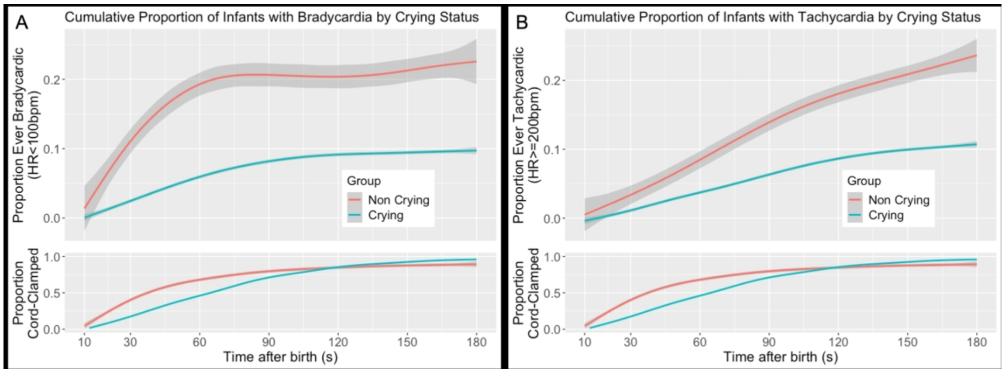

Cumulative proportion of neonates with a) bradycardia (heart rate <100 bpm for ≥1 seconds) and b) tachycardia (heart rate ≥200 bpm for ≥1 seconds) among non-crying but breathing and crying groups during first 180 second after birth with 95 % confidence intervals. Data were sampled every second and curves generated using a generalized additive model.

333x121mm (300 x 300 DPI)

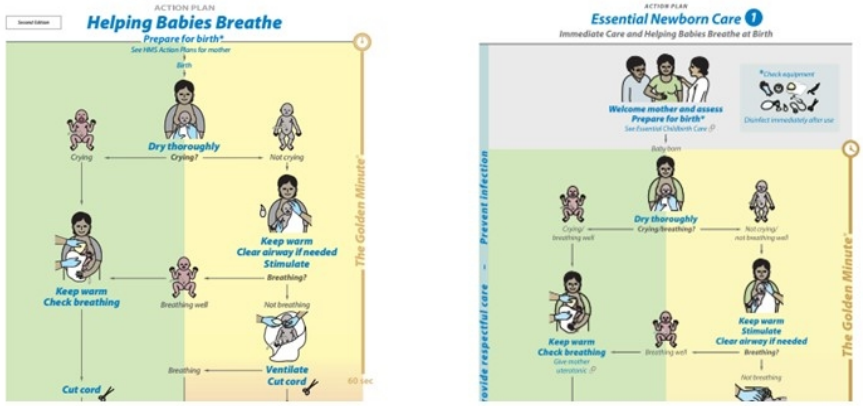

Preprinted with permission from Helping Babies Breathe Action Plan, 2nd edition, © 2016 American Academy of Pediatrics

Essential Newborn Care 1 Action Plan, © 2022 World Health Organization

210x100mm (96 x 96 DPI)

Supplementary tables

Supplementary Table 1. Details of obstetric complications among crying and non-crying but breathing neonates

Frequency of complications	Crying (N=1155)	%	Non crying but breathing (N=54)	%
Zero	1119	96.9	50	92.6
One	35	3.0	4	7.4
Two	1	0.1	0	0.0
Maternal conditions				
Hypothyroidism	1	0.1	0	0.0
Diabetes Mellitus inc. gestational diabetes	0	0.0	0	0.0
Oligohydramnios	1	0.1	1	1.9
Polyhydramnios	0	0.0	0	0.0
Delivery complications				
Antepartum hemorrhage	1	0.1	0	0.0
Pre-eclampsia	1	0.1	0	0.0
Eclampsia	0	0.0	0	0.0
Intrapartum fever	1	0.1	0	0.0
Chorioamnionitis	1	0.1	0	0.0
Premature rupture of membranes	4	0.3	0	0.0
Prolonged labour	12	1.0	2	3.7

Confidential: For Review Only

Decreased fetal movements	3	0.3	0	0.0
Breech or transverse lie	4	0.3	0	0.3
Prolapsed cord	1	0.1	0	0.0
Fetal distress	3	0.3	1	1.9
Maternal distress	1	0.1	0	0.0
Fetal/neonatal conditions				
Fetal congenital anomaly	4	0.3	0	0.0
Nuchal cord	0	0.0	0	0.0
Cephalopelvic disproportion	0	0.0	0	0.0

Confidential: For Review Only

Supplementary Table 2. Comparison of median heart rate (bpm) between crying and non-crying but breathing neonates. P values of < 0.004 were considered significant (p=0.05/12) using individual Mann-Whitney U-tests followed by *post hoc* Bonferroni correction for multiple comparisons.

	Crying		Non-crying but breathing		
Time	Median (quartiles)	N	Median (quartiles)	N	p-value
10 sec	157 (118, 174)	171	155 (104, 178)	5	0.929
30 sec	168 (146, 182)	988	164 (140, 173)	44	0.082
45 sec	167 (149, 182)	1099	165 (138, 182)	51	0.508
60 sec	168 (151, 182)	1106	162 (149, 185)	51	0.851
75 sec	169 (154, 182)	1044	168 (150, 187)	52	0.860
90 sec	169 (154, 183)	899	164 (156, 188)	50	0.559
105 sec	169 (155, 184)	794	175 (164, 190)	46	0.037
120 sec	169 (156, 183)	695	174 (161, 191)	45	0.086
135 sec	169 (156, 183)	495	173 (153, 190)	30	0.637
150 sec	170 (156, 182)	364	172 (149, 192)	24	0.655
165 sec	169 (155, 182)	290	177 (156, 192)	18	0.224
180 sec	169 (156, 182)	210	170 (161, 195)	15	0.163

Confidential: For Review Only

BMJ Paediatrics Open

Observational study comparing heart rate in crying and non-crying but breathing infants at birth

Journal:	*BMJ Paediatrics Open*
Manuscript ID	bmjpo-2023-001886.R1
Article Type:	Original research
Date Submitted by the Author:	04-Mar-2023

Complete List of Authors:	Kukka, Antti; Uppsala universitet Disciplinary Domain of Medicine and Pharmacy, Women's and Children's Health; Region Gävleborg, Department of Pediatrics<br>Berkelhamer, Sara; University of Washington, Division of Neonatology, Department of Pediatrics,<br>Eilevstjønn, Joar; Laerdal Medical AS, Strategic Research<br>Wood, Thomas; University of Washington, Center on Human Development and Disability<br>Basnet, Omkar; Golden Community<br>KC, Ashish; Uppsala University, International Maternal and Child Health

Keywords:	Neonatology, Physiology, Resuscitation

SCHOLARONE™
Manuscripts

I, the Submitting Author has the right to grant and does grant on behalf of all authors of the Work (as defined in the below author licence), an exclusive licence and/or a non-exclusive licence for contributions from authors who are: i) UK Crown employees; ii) where BMJ has agreed a CC-BY licence shall apply, and/or iii) in accordance with the terms applicable for US Federal Government officers or employees acting as part of their official duties; on a worldwide, perpetual, irrevocable, royalty-free basis to BMJ Publishing Group Ltd ("BMJ") its licensees and where the relevant Journal is co-owned by BMJ to the co-owners of the Journal, to publish the Work in this journal and any other BMJ products and to exploit all rights, as set out in our licence.

The Submitting Author accepts and understands that any supply made under these terms is made by BMJ to the Submitting Author unless you are acting as an employee on behalf of your employer or a postgraduate student of an affiliated institution which is paying any applicable article publishing charge ("APC") for Open Access articles. Where the Submitting Author wishes to make the Work available on an Open Access basis (and intends to pay the relevant APC), the terms of reuse of such Open Access shall be governed by a Creative Commons licence – details of these licences and which Creative Commons licence will apply to this Work are set out in our licence referred to above.

Other than as permitted in any relevant BMJ Author's Self Archiving Policies, I confirm this Work has not been accepted for publication elsewhere, is not being considered for publication elsewhere and does not duplicate material already published. I confirm all authors consent to publication of this Work and authorise the granting of this licence.

Confidential: For Review Only

Observational study comparing heart rate in crying and non-crying but breathing infants at birth

Antti Kukka[1,2*] MD, MSc, Sara Berkelhamer[3*], MD, Joar Eilevstjønn[4], PhD, Thomas Ragnar Wood[5], B.M., B.Ch., PhD, Omkar Basnet[6], MSc, Ashish KC[1], MD, PhD

Affiliations:

[1]Department of Women's and Children's Health, Uppsala University, Sweden

[2]Department of Pediatrics, Region Gävleborg, Sweden

[3]Division of Neonatology, Department of Pediatrics, University of Washington, Seattle, USA

[4]Laerdal Medical, Stavanger, Norway

[5]Center on Human Development and Disability, University of Washington, Seattle, USA

[6]Golden Community, Jawagal, Nepal

*Contributed equally as co-first authors

Corresponding author: Antti Kukka, Department of Women's and Children's Health, Uppsala University, SE-751 85, Uppsala, Sweden, https://kbh.uu.se. Email address: antti.kukka@kbh.uu.se; Phone number: +46 721 811 448

Clinical trial registration number: ISRCTN18148368

Keywords: heart rate, neonatal, resuscitation

Confidential: For Review Only

Abstract

Background: Stimulating infants to elicit a cry at birth is common but could result in unnecessary handling. We evaluated heart rate in infants who were crying versus non-crying but breathing immediately after birth.

Methods: This was single-center observational study of singleton vaginally born infants at ≥33 weeks of gestation. Infants who were *crying* or *non-crying but breathing* within 30 seconds after birth were included. Background demographic data and delivery room events were recorded using tablet-based applications and synchronized with continuous heart rate data recorded by a dry-electrode electrocardiographic monitor. Heart rate centile curves for first three minutes of life were generated with piece wise regression analysis. Odds of bradycardia and tachycardia were compared using multiple logistic regression.

Results: 1155 crying and 54 non-crying but breathing neonates were included in the final analyses. There were no significant differences in the demographic and obstetric factors between the cohorts. Non-crying but breathing infants had higher rates of early cord clamping < 60 seconds after birth (75.9% vs 46.5%) and admission to the neonatal intensive care unit (13.0% vs 4.3%). There were no significant differences in median heart rates between the cohorts. Non-crying but breathing infants had higher odds of bradycardia (heart rate < 100 bpm, adjusted odds ratio 2.64; 95% Confidence Interval 1.34-5.17) and tachycardia (adjusted odds ratio 2.86; 95% Confidence Interval 1.50-5.47).

Conclusion: Infants who are quietly breathing but do not cry after birth have an increased risk of both bradycardia and tachycardia and admission to neonatal intensive care unit.

248 words

What's Known on This Topic

Stimulation of breathing infants to elicit a cry is a common practice in newborn deliveries. However, international resuscitation algorithms disagree on whether *crying* or *breathing* should guide providing stimulation.

What This Study Adds

Non-crying but breathing infants have increased risk of bradycardia, tachycardia and neonatal intensive care admission compared to crying infants.

How this study might affect research, practice or policy

While waiting for further studies assessing the role of stimulation in non-crying but breathing infants, these newborns should be monitored carefully for clinical deterioration.

Word Count: 2531

Confidential: For Review Only

Contributors statement:

Dr. Ashish KC designed the study, implemented, supervised and carried out the study and the data collection on site, reviewed data analysis and drafted the initial manuscript.

Dr. Sara Berkelhamer designed the study, reviewed data analysis and revised and edited the manuscript.

Dr. Antti Kukka designed the study, reviewed the data analysis and drafted the initial manuscript.

Dr. Joar Eilevstjønn and Dr. Thomas Ragnar Wood extracted and analyzed the heart rate data and drafted the initial manuscript.

Omkar Basnet implemented, supervised and carried out the study and the data collection on site, and extracted and analyzed the heart rate data.

All authors approved the final manuscript as submitted and agree to be accountable for all aspects of the work.

Confidential: For Review Only

INTRODUCTION

The transition to air-breathing at birth is a seminal physiological event essential to the onset of pulmonary gas exchange and postnatal survival [1]. This transition is usually accompanied by a spontaneous or elicited cry, a reassuring and readily observable sign for both health care providers and family. The clinical implication of a breathing infant who *does not cry* during the first minute after birth warrants evaluation as the globally important Helping Babies Breathe (HBB) neonatal resuscitation algorithm utilizes lack of cry alone to direct further actions in newborn resuscitation [2].

Lung fluids play a critical role in early fetal lung development, but represent an obstacle to lung expansion and gas exchange post-delivery [3,4]. Airway liquid clearance is primarily driven by the expansion of the chest wall during inspiration [5]. As compared to quiet tidal breathing, crying similarly to grunting creates greater peak inspiratory flow and a more complex biphasic expiratory volume pattern preventing backflow of liquid into the alveoli [5,6]. This pattern is associated with optimal volume redistribution from well aerated regions to those which are not as well aerated via pendelluft flow [5]. Crying is thus better suited to both rapid aeration and maintenance of functional residual capacity than tidal breathing [5].

Lung recruitment at birth also plays a key role in cardiovascular transition and pulmonary blood flow. Lung aeration increases arterial oxygen content with subsequent decrease in local pulmonary vascular resistance, while shift of liquid from the alveoli to perialveolar tissue triggers a neurally mediated global increase in pulmonary blood flow [7]. This transition is key to supporting cardiac output with increases in pulmonary venous return.

Confidential: For Review Only

With clamping of the umbilical cord and closure of right-to-left shunts, pulmonary venous return becomes the sole source of left intracardiac volume [7]. If the cord is clamped before the lung is aerated, clinical compromise and a fall in cardiac output can theoretically lead to bradycardia [4,8]. However, a trial of physiologically-based cord clamping following established lung aeration did not show a reduction in the rate of bradycardia in infants needing resuscitation when compared to early cord clamping [9].

For low resource settings, simple clinical signs are needed for identifying infants in need of resuscitation. We previously demonstrated higher risk of neonatal mortality among neonates who were not crying immediately after birth [10]. This observation was particularly true for non-breathing infants, but even non-crying but breathing newborns had an increased risk of predischarge mortality when compared with crying neonates (adjusted odds ratio (aOR) 12.3; 95% confidence interval (CI), 5.8–26.1) [10]. To further investigate the contribution of cry to the transitional physiology of breathing infants, we sought to compare the HR pattern in the first three minutes in infants classified as *crying* or *non-crying but breathing* immediately after birth.

METHODS

Design

This was a secondary analysis of a single center prospective observational study, which was conducted over five months (28th May to 28th October 2019) at Western Regional Hospital, Pokhara, Nepal [11]. The hospital has approximately 6500 births annually and is the public referral hospital of the province.

Confidential: For Review Only

Ethical approval was sought from the Nepal Health Research Council (no. 87/2018) and written informed consent obtained from the pregnant women before delivery. Declaration of Helsinki by the World Medical Association was adhered to.

Deliveries were attended by a nurse-midwife or a skilled birth attendant with on-call support from a general medical doctor, pediatrician and obstetrician when needed [12]. Providers were trained in REFINE, a HR-guided neonatal resuscitation protocol, including HBB 2nd edition supplemented by a half-day orientation on the use of continuous HR monitoring [12].

Participants

All live born vaginally delivered singleton neonates at or beyond 33 weeks of gestation or with birth weight > 2500 grams were eligible for this study.

Crying newborns were defined as those who cried within approximately 30 seconds after birth.

Non-crying but breathing newborns were defined as those who did not cry but were noted to be breathing within the same period.

All newborns who were non-crying and non-breathing after birth were excluded from this study.

Data collection and management

Research nurses not part of the clinical care team recorded delivery room observations including periods of breathing and time of cord clamping using a purpose-designed application (Liveborn, Laerdal Medical, Stavanger, Norway) [12]. Oxygen saturation data was not recorded. Crying status after birth, and maternal and neonatal demographics were recorded on a supplemental application (SUSTAIN). Cases where breathing status recorded on Liveborn application did not align with the crying status documented on SUSTAIN were reviewed by expert panel (KCA, SB, JE, OB) blinded

Confidential: For Review Only

to the HR data. Comments provided by research nurses and actions taken during resuscitation were taken into consideration. As a result of the review, four cases were re-classified from non-breathing to non-crying but breathing and 18 cases were changed to non-breathing and thereby excluded.

Following thorough drying, NeoBeat continuous HR monitor (Laerdal Global Health, Stavanger, Norway) was placed on the abdomen of the newborn [13]. NeoBeat uses a proprietary algorithm based on zero crossing count that averages HR over up to 12 RR intervals. The HR data from NeoBeat was streamed to and time-synced with data collected on the Liveborn application. Bluetooth connection issues or excessive movement of the newborn resulting in absent or highly inconsistent HR required exclusion of cases.

Information from the Liveborn and SUSTAIN apps were extracted separately and cleaned. Unique identification numbers provided for each participant facilitated synchronization of data for final analysis.

Data analysis

Demographics

For the final data analysis, we excluded participants' data when the birth was registered in the Liveborn app more than 10 seconds after actual birth, time of birth was not provided, or if there was too little (<30 seconds), too late (>60 seconds after birth) or too inconsistent HR data.

Maternal and infant demographics, mode of delivery, birth weight, small for gestational age status (birth weight $<10^{th}$ centile for gestational age), gestational age, sex and timing of cord clamping (early <60 seconds, late ≥60 seconds after birth) were collected from patient records. List of maternal and obstetric complications considered is presented in Supplementary Table 1.

Confidential: For Review Only

147 Means in the two cohorts were compared using independent samples t-test and medians by Wilcoxon signed ranked test. Proportions were compared by Pearson's Chi-square test.

Confidential: For Review Only

Heart Rate data

HR data for the crying and non-crying but breathing groups from 10 until 180 seconds after birth were analyzed using centile curves at 3rd, 10th, 25th, 50th, 75th, 90th and 97th centile. The curves were smoothed using piecewise cubic spline fitting. Median HRs at two serial time points were compared every 15 seconds using Mann Whitney U test with *post hoc* Bonferroni correction of p-values to adjust for repeated measures ($p=0.05/12 = p\leq 0.004$ for significance).

The OR of bradycardia and tachycardia were explored by logistic regression. Time of cord clamping, gestational age in weeks, birthweight in kilograms, and obstetric complications including abnormal fetal HR were included as covariates. Results are presented as aOR with 95% CI.

Cumulative proportion of neonates with bradycardia (HR <100 bpm for ≥1 seconds) and tachycardia (HR ≥200 bpm for ≥1 seconds) in the crying and non-crying but breathing groups were presented using a generalized additive model with 95% CI of continuous data recorded every second.

Analyses were performed using R version 4.1.2 (Vienna, Austria) and MATLAB R2021a (Mathworks Inc, Natick, MA).

Patient and Public Involvement

No patient involved.

RESULTS

During the study period, 3578 deliveries took place at the hospital of which 1028 were not assessed for eligibility. Of the 2550 births observed, Liveborn was not used in 368 neonates, 57 resulted in a fresh stillbirth and 34 neonates had a gestational age less than 33 weeks. Of the neonates observed

Confidential: For Review Only

with Liveborn, 882 were excluded due to issues with the HR data, LiveBorn application or gestational age. Among the 1209 neonates included in the final analyses, 1155 were classified as crying and 54 as non-crying but breathing immediately after birth (Fig. 1).

There was no significant difference in maternal age, parity or ethnicity in the two cohorts. Infants in the two groups had similar birth weight, gestational age, sex distribution and median Apgar scores at 1 and 5 minutes (Table 1). Obstetric complications during admission, abnormal fetal HR during labor and rates of instrumental delivery were also comparable (Supplementary Table 1).

Table 1: Demographic, obstetric and neonatal characteristics of crying and non-crying but breathing infants

**Indicators**	**Crying (N=1155)**	**Non crying but breathing (N=54)**	**p-value**
**Demographic factors**			
**Maternal Age,** mean ± SD	24.2 ± 4.3	24.1 ± 4.6	0.29
**Parity,** n (%)			0.30
Primipara	622 (53.9)	33 (61.1)	
Multipara	533 (46.1)	21 (38.9)	
**Ethnicity,** n (%)			0.45
Dalit	250 (21.6)	15 (27.8)	
Janajati	443 (38.4)	24 (44.4)	
Madhesi	13 (1.1)	0 (0.0)	
Muslim	24 (2.1)	1 (1.9)	
Brahmin/Chhetri	425 (36.8)	14 (25.9)	
Others	0 (0.0)	1 (0.9)	
**Pregnancy and delivery**			
**Obstetric Complications,** n (%)			0.09
No	1119 (96.9)	50 (92.6)	
Yes	36 (3.1)	4 (7.4)	
**Abnormal fetal HR during labor,** n (%)			0.47
No	1144 (99.0)	54 (100.0)	
Yes	11 (1.0)	0 (0.0)	

Confidential: For Review Only

**Mode of Delivery,** n (%)			0.13
Spontaneous Vaginal	1115 (96.5)	50 (92.6)	
Instrumental	40 (3.5)	4 (7.4)	
**Neonatal factors**			
**Birth Weight grams*,** mean±SD	3065.7±414.8	3047.2±410.5	0.55
**Gestational Age weeks**,** mean±SD	39.2±1.3	39.1±1.6	0.19
**Preterm <37 weeks**,** n (%)			0.56
No	1095 (96.1)	51 (94.4)	
Yes	45 (3.9)	3 (5.6)	
**Small for gestational age <10th centile ***,** n (%)			0.66
No	942 (82.8)	46 (85.2)	
Yes	195 (17.2)	8 (14.8)	
**Cord Clamping,** n (%)			<0.001
Early <60 seconds after birth	537 (46.5)	41 (75.9)	
Delayed ≥60 seconds after birth	618 (53.5)	13 (24.1)	
**Sex,** n (%)			0.86
Male	634 (54.9)	29 (53.7)	
Female	521 (45.1)	25 (46.3)	
**Apgar Score**, Median (IQR)			
At 1 min	7 (7-7)	6 (6-6)	0.46
At 5 min	8 (8-8)	7 (7-8)	0.68
Missing *=3, **15, ***19.			
Chi-square test, Independent samples t-test, non-parametric comparison of medians			

Non-crying but breathing infants had higher proportion of early cord clamping than crying infants (75.9% vs 46.5%, p<0.001) and were more often admitted to the neonatal intensive care unit (13.0% vs 4.3%, p=0.003) (Table 2).

Confidential: For Review Only

Table 2. Care of crying and non-crying but breathing infants

**Indicators**	**Crying N=1155**	**Non crying but breathing N=54**	**p-value**
**Positive Pressure Ventilation <3 min,** n (%)			0.76
No	1153 (99.8)	54 (100.0)	
Yes	2 (0.2)	0 (0.0)	
**Transfer to neonatal intensive care unit,** n (%)			0.003
No	1105 (95.7)	47 (87.0)	
Yes	50 (4.3)	7 (13.0)	
Chi-square test			

The smoothed 3rd, 10th, 25th, 50th, 75th, 90th and 97th HR centiles in crying and non-crying but breathing groups are displayed in Fig. 2(a) and (b). The 10th centile HR remained <100 bpm out to 35 and 20 seconds in the non-crying but breathing and crying cohorts, respectively. There were no significant differences in median HRs in the crying and non-crying but breathing cohorts between 10 and 180 seconds (Supplementary Table 2).

The aOR of bradycardia during the first 180 seconds after birth was increased in the non-crying but breathing cohort compared to the crying cohort (2.64; 95% CI 1.34-5.17; p=0.005). Similarly, the aOR of tachycardia was increased to 2.86 (95% CI 1.50-5.47, p=0.002) (Table 3).

Table 3. Adjusted Odds Ratio for experiencing bradycardia (heart rate <100 bpm) and tachycardia (heart rate ≥200 bpm) after cord clamping within the first 180 seconds after birth.

	**Bradycardia**	**Tachycardia**
Non-crying	**2.64 (1.34-5.17; p=0.005)**	**2.86 (1.50-5.47; p=0.002)**
Gestational age (per week)	0.98 (0.85-1.13; p=0.77)	1.06 (0.92-1.22; p=0.41)
Birthweight (per kg)	1.23 (0.77-1.99; p=0.38)	1.09 (0.69-1.73; p=0.71)
Obstetric complications	1.43 (0.73-2.80; p=0.30)	**3.43 (2.00-5.87; p<0.001)**
Early cord clamping (<60 seconds)	1.12 (0.77-1.64; p=0.55)	1.03 (0.72-1.48; p=0.86)

Confidential: For Review Only

Median (interquartile range) duration of bradycardia was 7 seconds (4-16 seconds) and tachycardia 11 seconds (6-25 seconds). A total of 6/54 (11.1%) and 48/1155 (4.2%) infants were ever bradycardic, and 6/54 (11.1%) and 71/1155 (6.1%) ever tachycardic in the non-crying and crying groups, respectively. Bradycardia and tachycardia largely affected separate infants – only one infant was both bradycardic and tachycardic within the first 180 seconds after birth.

A comparison of the cumulative proportion of infants with bradycardia and tachycardia in the crying and non-crying but breathing groups is presented Fig. 3 (a) and (b). Bradycardia appeared to be a relatively early phenomenon, with tachycardia appearing later, particularly in the non-crying but breathing group.

DISCUSSION

Crying confers physiological advantage over quiet breathing for clearance of lung liquids at birth. This single center observational study compared HR in 1155 crying and 54 non-crying but breathing neonates during the first three minutes after birth. There was no difference in the median HR between the two groups, but a larger proportion of non-crying but breathing neonates displayed unstable HR pattern of either bradycardia (aOR 2.64, 95% CI 1.34-5.17; p=0.005) or tachycardia (aOR 2.86, 95% CI 1.50-5.47, p=0.002) and higher risk of admission to neonatal intensive care unit.

Previous papers have presented centile curves for neonatal HR [9,14–16]. Crying infants were similar to term vaginally born cohort described by Bjorland et al. [15], but non-crying but breathing infants were more likely to remain bradycardic and experience delayed tachycardia. Prior studies have identified an increased risk of bradycardia among preterm neonates [14], however the distribution of gestational age and birth weight did not differ between the crying and non-crying

Confidential: For Review Only

but breathing groups in our study. Timing of cord clamping also impacts HR with early clamping increasing the risk of bradycardia [16,17]. In this study, non-crying but breathing neonates were more likely to undergo early cord clamping than crying neonates (75.9% vs 46.5%), but the difference in odds of bradycardia remained statistically significant after this and other adjustments. Early cord clamping may, however, explain some of the differences in timing of bradycardia observed. Furthermore, the clinicians' decision to perform early cord clamping might have been impacted by the lack of early cry or the observed HR.

Another cause of bradycardia in newborns is hypoxia-induced vagal reflex [18]. This phenomenon has been shown most convincingly in relation to apnoea in the setting of obstructed placental blood flow [19], but as newborns who do not cry at birth have slower clearance of liquid from airways [5], it is conceivable that even non-crying but breathing neonates could have lower oxygenation than crying neonates. Unfortunately, we were not able to measure peripheral saturation during the study to verify this potential explanatory mechanism. Lack of pulse oximeters is a common limitation in low-income settings and until their availability increases, caregivers need actionable clinical signs like crying to guide their resuscitation efforts.

Results of this study have potential implications for neonatal resuscitation algorithms and clinical practice. Current resuscitation guidelines are inconsistent in whether breathing or crying should guide the need for resuscitation (Supplementary Fig 1). The HBB algorithm uses crying as an indicator that the infant is breathing well enough to not need stimulation [2]. In contrast, to avoid unnecessary handling the recently released World Health Organization Essential Newborn Care 1 algorithm suggests that if an infant is either crying or breathing well then stimulation is not required [20].

https://mc.manuscriptcentral.com/bmjpo

Differentiating well breathing from less effective respiratory effort is challenging and saturation measurement or abdominal breathing monitoring are not always available in low-income settings. Findings of this study, along with previous research showing higher odds of mortality among non-crying but breathing infants as compared to those who were crying at birth [10], suggest that the role of stimulation in non-crying but breathing neonates be examined in a randomized trial. However, such study would be hard to conduct as the proportion of non-crying but breathing infants is low.

Strengths

Outcome data of the study was measured with NeoBeat HR monitor that has been shown to record efficient and reliable HR data [13]. Trained external data collectors directly observed all births and classified crying status immediately after stabilization [11,12,21]. To further increase reliability of the crying classification, we cross-tabulated data from breathing observations made real time during the initial stabilization and found high rates of agreement. Any potential misclassification might nonetheless have created spurious associations as the number of non-crying but breathing infants was relatively small.

Limitations

Evaluating breathing patterns in a newborn can be difficult and complex systems utilizing flow measurement, video and audio recordings are required for confident classification [5]. In this study, crying and breathing status at birth were determined based on overall impression during the first 30 seconds after birth with unclear interobserver reliability and risk of bias compared with video recording. The results should therefore be considered preliminary and advocate for validation with a video review. We were also unable to separate any form of breathing from

Confidential: For Review Only

breathing well, which might have biased the non-crying but breathing cohort towards sicker babies than what is intended with the Essential Newborn Care 1 resuscitation algorithm [20]. The same classification issue is, however, encountered by practitioners trying to apply the algorithm to clinical practice. Exclusion of circa 15 % of babies born with caesarean section limits the generalizability of the findings [12]. This study was underpowered to explore differences in outcome beyond admission to neonatal intensive care unit. Lastly, the potential relationship between non-crying and hypoxia as the explanatory mechanism for bradycardia and tachycardia could not be defined due to lack of concomitant oxygen saturation measurement. Further studies allowing recorded review for classification of both crying and breathing status coupled with simultaneous HR and saturation measurement should be performed in a sufficiently large population group to provide definitive guidance.

CONCLUSIONS

Crying is a simple clinical indicator of likely adequate lung recruitment facilitating normal physiologic transition at birth. Infants who are breathing but *do not cry* are at increased risk of cardiopulmonary instability and should therefore be monitored closely.

Confidential: For Review Only

Acknowledgements: We would like to thank research nurses, pediatricians and nurses in labor and delivery unit at Pokhara Academy of Health Sciences. We would like to thank Ram Chandra Bastola and Pratiksha Bhattarai for the clinical advice and Nick Brown for his comments on the manuscript.

Funding: Laerdal Foundation for Acute Medicine, Norway (2019-40499) and Grand Challenges Canada (1910-30925).

Role of funder: The external funding sources had no role in study design, data collection, data analysis, data interpretation, writing of the report or in the decision to submit the paper for publication.

Conflict of interest: JE is employed at Laerdal Medical. AK received PhD funding via grant Laerdal Foundation grant given to Uppsala University. All other authors had no other financial relationships with any organizations that might have an interest in the submitted work in the previous 3 years or no other relationships or activities that could appear to have influenced the submitted work.

Confidential: For Review Only

REFERENCES

1 Hooper SB, Polglase GR, Roehr CC. Cardiopulmonary changes with aeration of the newborn lung. *Paediatric respiratory reviews* 2015;**16**:147–50. doi:10.1016/j.prrv.2015.03.003

2 Kamath-Rayne BD, Thukral A, Visick MK, *et al.* Helping Babies Breathe, Second Edition: A Model for Strengthening Educational Programs to Increase Global Newborn Survival. *Global Health: Science and Practice* 2018;**6**:538–51. doi:10.9745/GHSP-D-18-00147

3 Gao Y, Raj JU. Regulation of the pulmonary circulation in the fetus and newborn. *Physiological reviews* 2010;**90**:1291–335. doi:10.1152/physrev.00032.2009

4 Hooper SB, Polglase GR, Te Pas AB. A physiological approach to the timing of umbilical cord clamping at birth. *Archives of Disease in Childhood-Fetal and Neonatal Edition* 2015;**100**:F355–60. doi:10.1136/archdischild-2013-305703

5 Tingay DG, Farrell O, Thomson J, *et al.* Imaging the Respiratory Transition at Birth: Unraveling the Complexities of the First Breaths of Life. *Am J Respir Crit Care Med* 2021;**204**:82–91. doi:10/gn7nws

6 te Pas AB, Wong C, Kamlin COF, *et al.* Breathing patterns in preterm and term infants immediately after birth. *Pediatric Research* 2009;**65**:352–6. doi:10.1203/PDR.0b013e318193f117

7 Hooper SB, Roberts C, Dekker J, *et al.* Issues in cardiopulmonary transition at birth. *Seminars in Fetal and Neonatal Medicine* 2019;**24**:101033. doi:10.1016/j.siny.2019.101033

8 Bhatt S, Alison BJ, Wallace EM, *et al.* Delaying cord clamping until ventilation onset improves cardiovascular function at birth in preterm lambs. *The Journal of physiology* 2013;**591**:2113–26. doi:10.1113/jphysiol.2012.250084

9 Badurdeen S, Davis PG, Hooper SB, *et al.* Physiologically based cord clamping for infants ≥32+0 weeks gestation: A randomised clinical trial and reference percentiles for heart rate and oxygen saturation for infants ≥35+0 weeks gestation. *PLoS Med* 2022;**19**:e1004029. doi:10.1371/journal.pmed.1004029

10 Ashish KC, Lawn JE, Zhou H, *et al.* Not Crying After Birth as a Predictor of Not Breathing. *Pediatrics* 2020;**145**:e20192719. doi:10.1542/peds.2019-2719

11 Gurung R, Gurung A, Basnet O, *et al.* REFINE (Rapid Feedback for quality Improvement in Neonatal rEsuscitation): an observational study of neonatal resuscitation training and practice in a tertiary hospital in Nepal. *BMC Pregnancy Childbirth* 2020;**20**:1–6. doi:10.1186/s12884-020-03456-z

12 Bhattarai P, Gurung R, Basnet O, *et al.* Implementing quality improvement intervention to improve intrapartum fetal heart rate monitoring during COVID-19 pandemic-observational study. *PloS one* 2022;**17**:e0275801. doi:10.1371/journal.pone.0275801

Confidential: For Review Only

13 Pike H, Eilevstjønn J, Bjorland P, *et al.* Heart rate detection properties of dry-electrode ECG compared to conventional 3-lead gel-electrode ECG in newborns. *BMC research notes* 2021;**14**:1–5. doi:10.1186/s13104-021-05576-x

14 Dawson JA, Kamlin COF, Wong C, *et al.* Changes in heart rate in the first minutes after birth. *Archives of Disease in Childhood-Fetal and Neonatal Edition* 2010;**95**:F177–81. doi:10.1136/adc.2009.169102

15 Bjorland PA, Ersdal HL, Eilevstjønn J, *et al.* Changes in heart rate from 5 s to 5 min after birth in vaginally delivered term newborns with delayed cord clamping. *Archives of Disease in Childhood - Fetal and Neonatal Edition* Published Online First: 10 November 2020. doi:10.1136/archdischild-2020-320179

16 Padilla-Sánchez C, Baixauli-Alacreu S, Cañada-Martínez AJ, *et al.* Delayed vs immediate cord clamping changes oxygen saturation and heart rate patterns in the first minutes after birth. *The Journal of Pediatrics* 2020;**227**:149-156. e1. doi:10.1016/j.jpeds.2020.07.045

17 Kc A, Kong SYJ, Haaland SH, *et al.* Increased risk of bradycardia in vigorous infants receiving early as compared to delayed cord clamping at birth. *J Perinatol* Published Online First: 31 December 2022. doi:10.1038/s41372-022-01593-1

18 Te Pas AB, Sobotka K, Hooper SB. Novel Approaches to Neonatal Resuscitation and the Impact on Birth Asphyxia. *Clinics in Perinatology* 2016;**43**:455–67. doi:10.1016/j.clp.2016.04.005

19 Dawes GS, Jacobson HN, Mott JC, *et al.* The treatment of asphyxiated, mature foetal lambs and rhesus monkeys with intravenous glucose and sodium carbonate. *The Journal of physiology* 1963;**169**:167–84. doi:10.1113/jphysiol.1963.sp007248

20 World Health Organization. WHO recommendations on newborn health: guidelines approved by the WHO Guidelines Review Committee. 2017.https://apps.who.int/iris/rest/bitstreams/1090525/retrieve (accessed 9 Jun 2022).

21 and EN-BIRTH Study Group, Kc A, Peven K, *et al.* Neonatal resuscitation: EN-BIRTH multi-country validation study. *BMC Pregnancy Childbirth* 2021;**21**:235. doi:10.1186/s12884-020-03422-9

Confidential: For Review Only

Supplementary material

Supplementary Table 1. Details of obstetric complications among crying and non-crying but breathing neonates

Supplementary Table 2. Comparison of median heart rate (bpm) between crying and non-crying but breathing neonates. P values of < 0.004 were considered significant (p=0.05/12) using individual Mann-Whitney U-tests followed by *post hoc* Bonferroni correction for multiple comparisons.

Supplementary Figure: Comparison of the Helping Babies Breathe 2nd edition and the Essential Newborn Care Now resuscitation algorithms.

Confidential: For Review Only

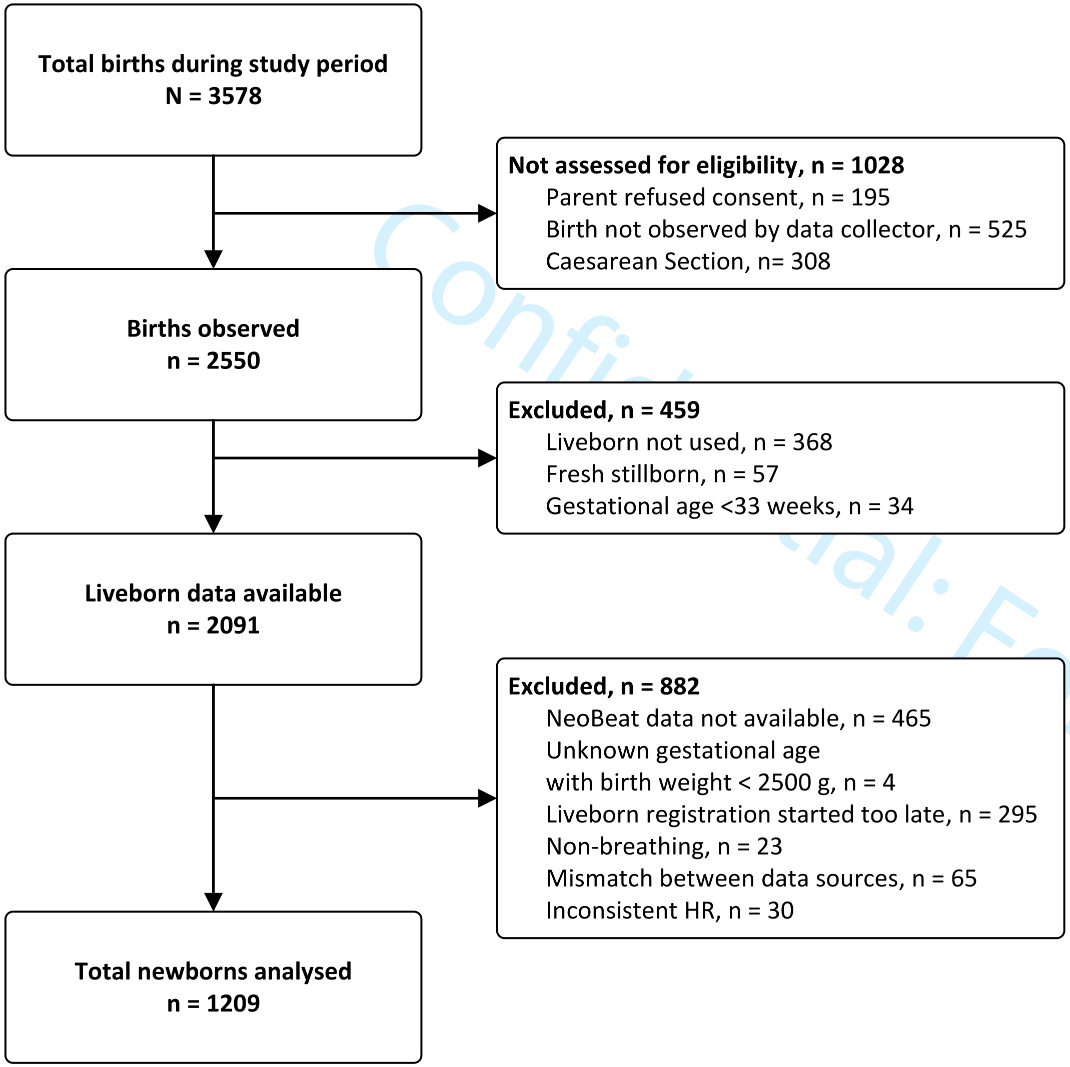

Confidential: For Review Only

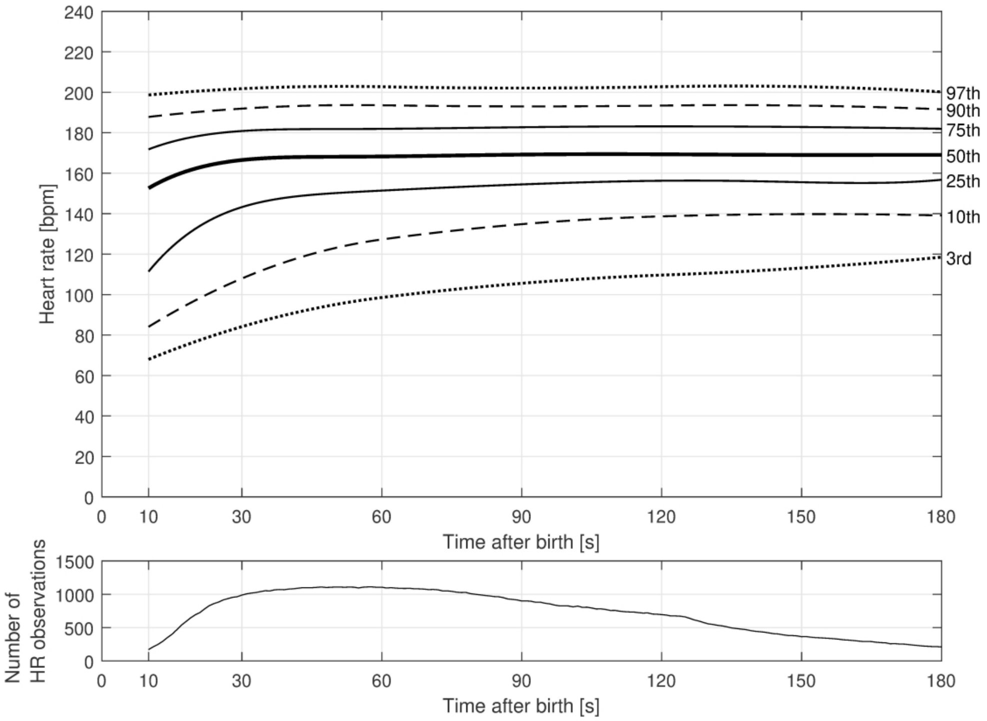

Figure 2a: Smoothed heart rate percentiles for crying infants during the first 180 seconds after birth

198x148mm (600 x 600 DPI)

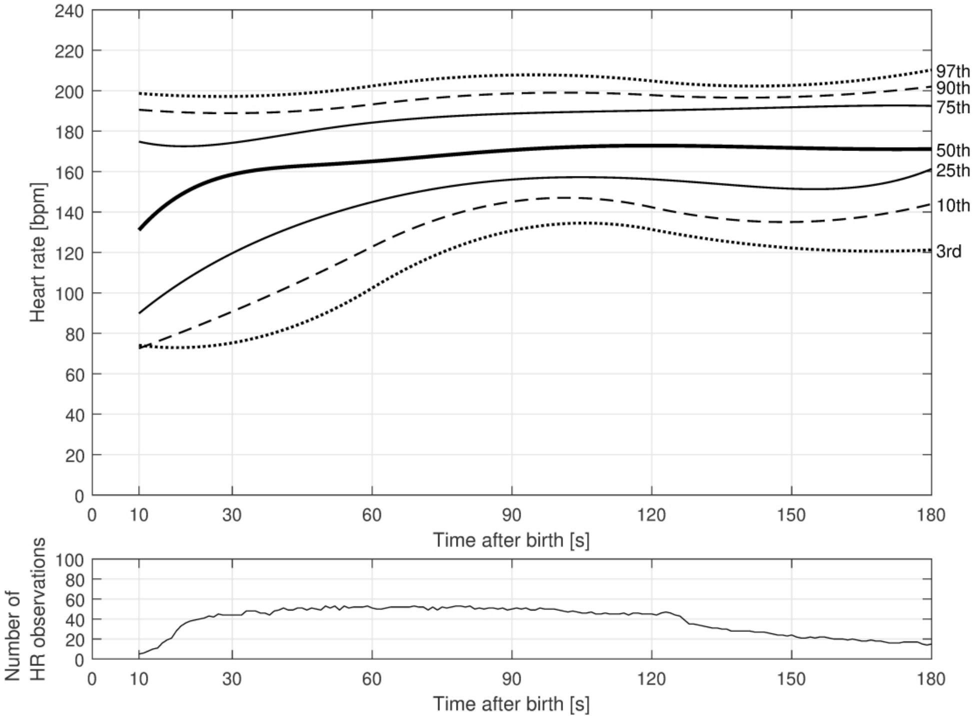

Figure 2b: Smoothed heart rate percentiles for non-crying but breathing infants during the first 180 seconds after birth

198x148mm (600 x 600 DPI)

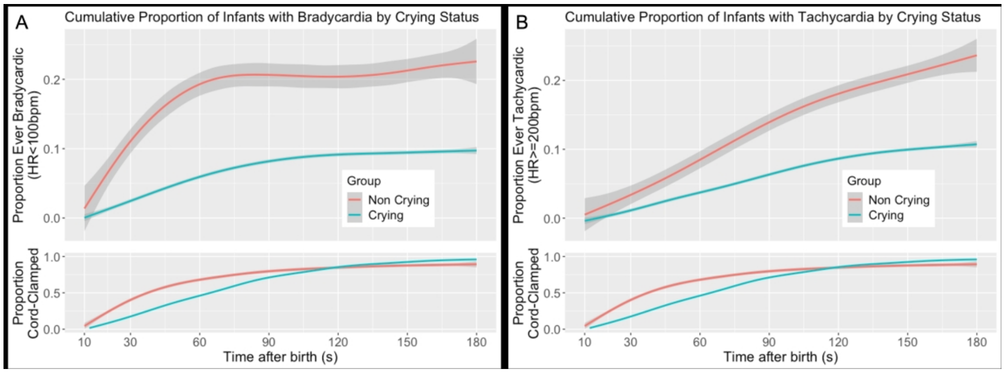

Cumulative proportion of neonates with a) bradycardia (heart rate <100 bpm for ≥1 seconds) and b) tachycardia (heart rate ≥200 bpm for ≥1 seconds) among non-crying but breathing and crying groups during first 180 second after birth with 95 % confidence intervals. Data were sampled every second and curves generated using a generalized additive model.

333x121mm (330 x 330 DPI)

SUPPLEMENTARY FILE

Supplementary tables

Supplementary Table 1. Details of obstetric complications among crying and non-crying but breathing neonates

**Frequency of complications**	**Crying (N=1155)**	**%**	**Non crying but breathing (N=54)**	**%**
Zero	1119	96.9	50	92.6
One	35	3.0	4	7.4
Two	1	0.1	0	0.0
Maternal conditions				
Hypothyroidism	1	0.1	0	0.0
Diabetes Mellitus inc. gestational diabetes	0	0.0	0	0.0
Oligohydramnios	1	0.1	1	1.9
Polyhydramnios	0	0.0	0	0.0
Delivery complications				
Antepartum hemorrhage	1	0.1	0	0.0
Pre-eclampsia	1	0.1	0	0.0
Eclampsia	0	0.0	0	0.0
Intrapartum fever	1	0.1	0	0.0
Chorioamnionitis	1	0.1	0	0.0
Premature rupture of membranes	4	0.3	0	0.0
Prolonged labour	12	1.0	2	3.7
Decreased fetal movements	3	0.3	0	0.0
Breech or transverse lie	4	0.3	0	0.3
Prolapsed cord	1	0.1	0	0.0
Fetal distress	3	0.3	1	1.9
Maternal distress	1	0.1	0	0.0
Fetal/neonatal conditions				
Fetal congenital anomaly	4	0.3	0	0.0
Nuchal cord	0	0.0	0	0.0
Cephalopelvic disproportion	0	0.0	0	0.0

Confidential: For Review Only

Supplementary Table 2. Comparison of median heart rate (bpm) between crying and non-crying but breathing neonates. P values of < 0.004 were considered significant (p=0.05/12) using individual Mann-Whitney U-tests followed by *post hoc* Bonferroni correction for multiple comparisons.

	Crying		Non-crying but breathing		
Time	Median (quartiles)	N	Median (quartiles)	N	p-value
10 sec	157 (118, 174)	171	155 (104, 178)	5	0.929
30 sec	168 (146, 182)	988	164 (140, 173)	44	0.082
45 sec	167 (149, 182)	1099	165 (138, 182)	51	0.508
60 sec	168 (151, 182)	1106	162 (149, 185)	51	0.851
75 sec	169 (154, 182)	1044	168 (150, 187)	52	0.860
90 sec	169 (154, 183)	899	164 (156, 188)	50	0.559
105 sec	169 (155, 184)	794	175 (164, 190)	46	0.037
120 sec	169 (156, 183)	695	174 (161, 191)	45	0.086
135 sec	169 (156, 183)	495	173 (153, 190)	30	0.637
150 sec	170 (156, 182)	364	172 (149, 192)	24	0.655
165 sec	169 (155, 182)	290	177 (156, 192)	18	0.224
180 sec	169 (156, 182)	210	170 (161, 195)	15	0.163

Confidential: For Review Only

Supplementary Figure. Comparison of the first minute of action in the Helping Babies Breathe 2nd edition (left) and Essential Newborn Care 1 (right) neonatal resuscitation flow-charts.

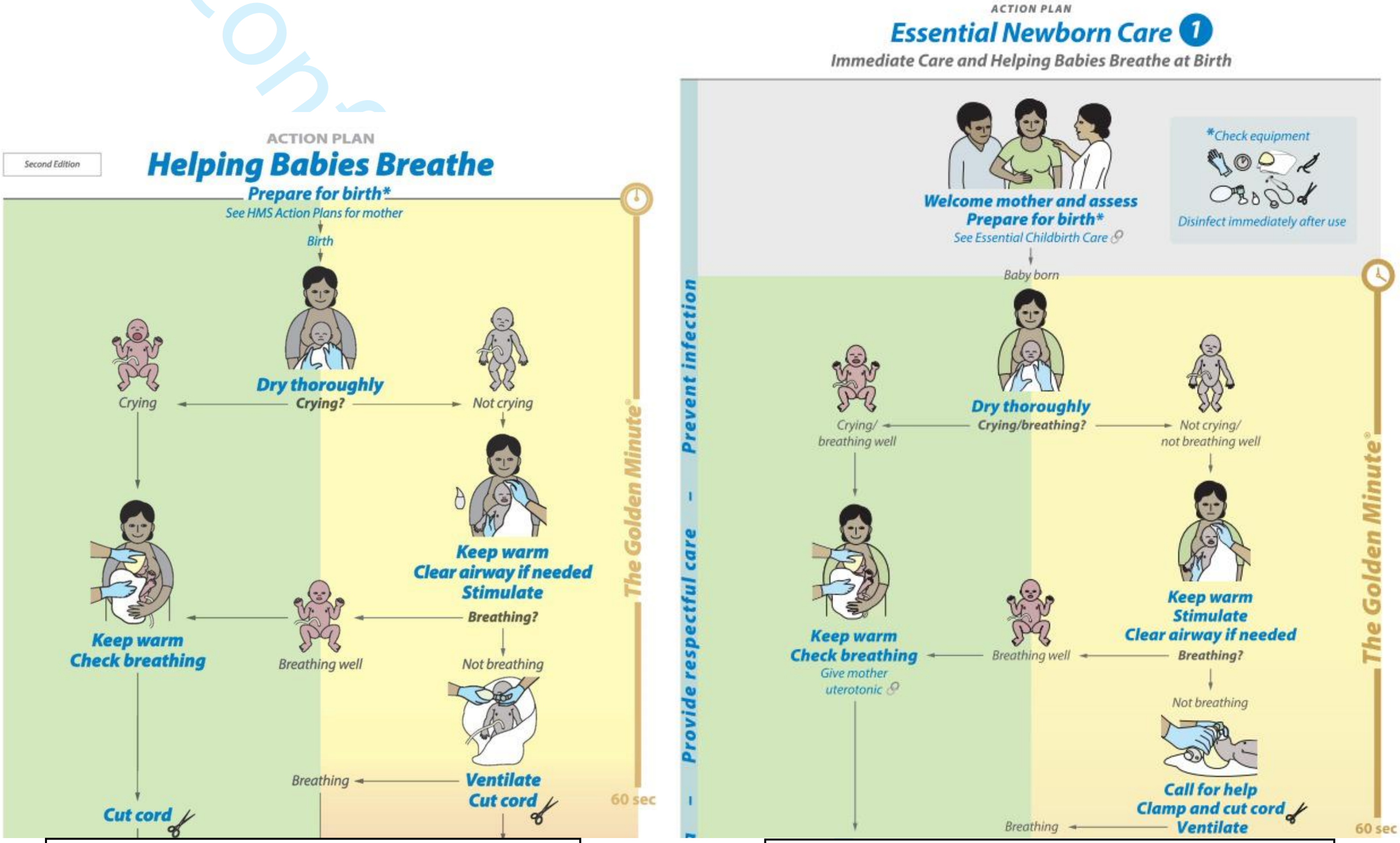

Printed with permission from Helping Babies Breathe Action Plan, 2nd edition, © 2016, American Academy of Pediatrics

Essential Newbon Care 1 Action Plan, © 2022, World Health Organization
